# ^1^H NMR metabolic phenotyping of *Dipterocarpus alatus* as a novel tool for age and growth determination

**DOI:** 10.1371/journal.pone.0243432

**Published:** 2020-12-15

**Authors:** Jutarop Phetcharaburanin, Suthicha Deewai, Thanaporn Kulthawatsiri, Komkid Moolpia, Manida Suksawat, Bundit Promraksa, Poramate Klanrit, Nisana Namwat, Watcharin Loilome, Kitisak Poopasit, Somporn Katekaew, Penprapa Phetcharaburanin

**Affiliations:** 1 Department of Biochemistry, Faculty of Medicine, Khon Kaen University, Khon Kaen, Thailand; 2 Cholangiocarcinoma Research Institute, Faculty of Medicine, Khon Kaen University, Khon Kaen, Thailand; 3 Khon Kaen University International Phenome Laboratory, Northeastern Science Park, Khon Kaen University, Khon Kaen, Thailand; 4 Center of Excellence for Innovation in Chemistry, Faculty of Science, Khon Kaen University, Khon Kaen, Thailand; 5 Department of Biochemistry, Faculty of Science, Khon Kaen University, Khon Kaen, Thailand; 6 Museum and Lifelong Learning Center, Khon Kaen University, Khon Kaen, Thailand; 7 Department of Chemistry, Faculty of Science, Khon Kaen University, Khon Kaen, Thailand; 8 Department of Environmental Science, Faculty of Science, Khon Kaen University, Khon Kaen, Thailand; 9 Coordination Center of the Royal Initiative Projects, Khon Kaen University, Khon Kaen, Thailand; CIC bioGUNE, SPAIN

## Abstract

*Dipterocarpus alatus* belongs to Family Dipterocarpaceae that can be commonly found in Southeast Asian countries. It is a perennial plant with oval-shaped leaves and oleoresin-rich wood. It has been considered as a multipurpose plant since all parts can be practically utilized. One of the major problems for utilizing *Dipterocarpus alatus* is the difficulty knowing the exact age as this kind of plant is ready for multipurpose use after 20 years of age. At present, the most commonly used method for determining age of *Dipterocarpus alatus* is the annual ring estimation. However, this conventional method is unable to provide the high precision and accuracy of age determination due to its limitation including blurry annual rings caused by enriched oleoresin in the wood. The current study aimed to investigate the differences of ^1^H -NMR spectroscopy-based metabolic profiles from bark and leaf of *Dipterocarpus alatus* at different ages including 2, 7, 15 and 25 years. Our findings demonstrated that there is a total of 56 metabolites shared between bark and leaf. It is noticeable that bark at different ages exhibited the strongest variation and sugar or sugar derivatives that were found in higher concentrations in bark compared with those in leaf. We found that decreasing levels of certain metabolites including tagatose, 1’kestose and 2’-fucosyllactose exhibited the promising patterns. In conclusion, panel metabolites involved in the sucrose biosynthesis can precisely determine the age and growth of *Dipterocarpus alatus*.

## Introduction

*Dipterocarpus alatus* belongs to Family Dipterocarpaceae that can be commonly found in Southeast Asian countries, especially Thailand, Cambodia, Lao, Vietnam, and Philippines [1]. *Dipterocarpus alatus* is a perennial plant with oval-shaped leaves and oleoresin-rich wood [2]. This tree is considered as a multipurpose plant since all parts can be economically utilized. One of the most utilized parts is resin which can be mainly used as the alternative energy fuel for agricultural diesel engine [3]. Previously, several studies showed that utilization of *Dipterocarpus alatus* also involves ecological aider which helps control air temperature, prevent soil erosion and encourage growth of fungi and mycorrhiza [3]. In regards with economic aspect, *Dipterocarpus alatus* can be made into plywood and exported to foreign countries. In addition, resins can be used for local livelihoods and exports [4]. Moreover, medicinal use of *Dipterocarpus alatus* involves bioactive compounds that possess the prevention and/or treatment properties such as anti-cancer, anti-bacterial, anti-human immunodeficiency virus, anti-inflammatory and treatment properties [5–7]. In cosmetics, its bioactive compounds are used as the components in perfume and make-up foundation products [8, 9].

One of the major concerns prior to the use of *Dipterocarpus alatus* is the age estimation as only the trees aged 25 years and above are usable. Despite several previous studies reporting a wide spectrum of phytochemicals obtained from different parts of *Dipterocarpus alatus* for pharmaceutical uses [10], there is, so far, no such reports exhibiting the age- or growth-associated metabolites of *Dipterocarpus alatus*. The current scientific method commonly used for age estimation is a physical method employing the calculation of the trunk diameter with growth factors and annual ring drilling [11]. However, there is a huge problem encountered against the unclear annual rings caused by rich-oleoresin wood. Hence, an alternative method for age and growth estimation is still needed. Metabolomics is the quantitative and qualitative measurement of metabolites in a biological system and is the latest omics suit in systems biology [12–15] that has been applied, in the current study, to determine the age and growth through the detection of changing metabolites in different parts of *Dipterocarpus alatus*.

## Materials and methods

### Plant collection

Leaves and barks of *Dipterocarpus alatus* aged 2, 7, 15 and 25 years (n = 5 each group) were harvested from *Dipterocarpus alatus* field of Khon Kaen University (KKU), Khon Kaen, Thailand (16°26'54.9"N 102°49'00.4"E) in June 2019. The KKU plantation field employed in the current study is generally composed of sandy-textured saline soils that is similar to other parts of northeastern region of Thailand. Taxonomic classification of plant was conducted by Asst. Prof. Dr. Penprapa Phetcharaburanin (Department of Environmental Science, Faculty of Science, Khon Kaen University).

### Circumference and stature measurements

Circumference and stature of *Dipterocarpus alatus* aged 2, 7, 15 and 25 years from *Dipterocarpus alatus* field of Khon Kaen University, Khon Kaen, Thailand were measured using measuring tape and Haga altimeter, respectively.

### Chemicals and reagents

Potassium dihydrogen phosphate (KH_2_PO_4_) and Deuterium Oxide (D_2_O) were purchased from Merck (Darmstadt, Germany). 3-(Trimethyl-silyl)propionic acid-d4 sodium salt (TSP), used as the internal standard reference for NMR analysis, was purchased from Cambridge Isotope Laboratories (Andover, MA, USA).

### Sample preparation and crude extraction

Leaves and barks of *Dipterocarpus alatus* were dried in a hot air oven at 50˚C. The dried leaves and barks were chopped and mashed into fine powder (100 g). Next, the powder was macerated in one liter of 90% ethanol for five days at room temperature. The liquid extract was evaporated under a vacuum using a rotary evaporator (Buchi, Switzerland). The crude extracts of leaves and barks were separately obtained and stored at 4 ˚C for further analyses [16].

### ^1^H-NMR-based metabolic profiling

Twenty mg of plant extract was placed into a test tube. D_2_O (pH 6.0) containing 0.1% (w/w) TSP was added and vortexed for 1 min at room temperature. After 20 min ultrasonication at room temperature, the mixture was passed through a 0.20 μm filter (Corning, USA). The supernatant was transferred into a 5 mm NMR tube. Proton NMR spectra were acquired using a 400 MHz NMR spectrometer (Bruker, USA). A Carr−Purcell−Meiboom−Gill (CPMG) pulse sequence [RD−90°−(ꞇ−180°−ꞇ)n−acquisition] was applied to samples at 310 K (2ꞇn = 76.8ms) in order to improve the visualization of signals generated from low molecular weight metabolites. A total of 64 scans were recorded into 72 k data points with a spectral width of 20 ppm. Chemical shift referencing, baseline correction and phasing were performed. Next, the NMR spectral data were processed using MestReNova (Mestrelab Research, USA) software to adjust the peak alignment and normalization using probabilistic quotient normalization. To confirm the assignment of correlated resonances, statistical total correlation spectroscopy (STOCSY) was employed. Moreover, the resonances of interest were searched against online metabolite databases such as biological magnetic resonance data bank (BMRD) and human metabolome database (HMDB) [16]. Metabolite list is present in S1 Table in S1 File.

### Multivariate statistical analysis of metabolome data

Processed spectral data matrix was imported to SIMCA version 14.1 (Umetrics Inc., Sweden) to conduct principal component analysis (PCA) with a pareto scaling method to visualize metabolic similarities and differences and to identify possible outliers, followed by an orthogonal signal correction-projection to latent structures (O-PLS) and O-PLS discriminant analysis (O-PLS-DA) in MATLAB R2016a (MathWorks, USA) environment. MATLAB was used to generate O-PLS-DA scores and coefficient plots, with a color visualization of correlation values |r| of each variable. Red color indicates higher correlation whilst blue indicates lower correlation of the variables with the classification. The fitness and predictability of the models obtained from OPLS-DA were determined by the R^2^ and Q^2^ values, respectively. Model validity was determined by CV-ANOVA p-value of each model. O-PLS and O-PLS-DA models in the current study were constructed based on one PLS component and one orthogonal component using mean-centered and pareto-scaled spectral data sets. Circumference and stature from all groups were statistically correlated with leaf and bark metabolic profiles using O-PLS regression analysis. The validation of all O-PLS-DA and O-PLS models involved in this study was assessed using CV-ANOVA *p*-value (*p* < 0.05) [17–19].

### Quantification of absolute concentration and multiple comparison test

Maximum intensity of selected metabolites was obtained for quantification of absolute concentration in regards with known concentration of TSP using the formula below:

$$Absolute\;concentration=\frac{Number\;of\;proton\;of\;TSP}{Number\;of\;proton\;of\;peak\;signal}\;x\;\frac{Maximum\;intensity\;of\;peak\;signal}{Maximum\;intensity\;of\;TSP}xConcentration\;of\;TSP$$

Tukey’s multiple comparison test was performed on the absolute concentration using GraphPad Prism 8.0.1 (GraphPad Software, USA) that uses pairwise post-hoc testing to determine whether there is a difference between the mean of all possible pairs using a studentized range distribution.

## Results

### Circumference and stature of *Dipterocarpus alatus* at different ages

In the current study, we have measured both circumference and stature of *Dipterocarpus alatus* at 2, 7, 15 and 25 years of age, planted on the same field at Khon Kaen University with known dates of plantation. These parameters were used as the basal information that was further integrated with metabolome data obtained from both bark and leaf in order to investigate age- or growth-dependent metabolite fingerprints that can be applied for age or growth estimation.

**Fig 1:**
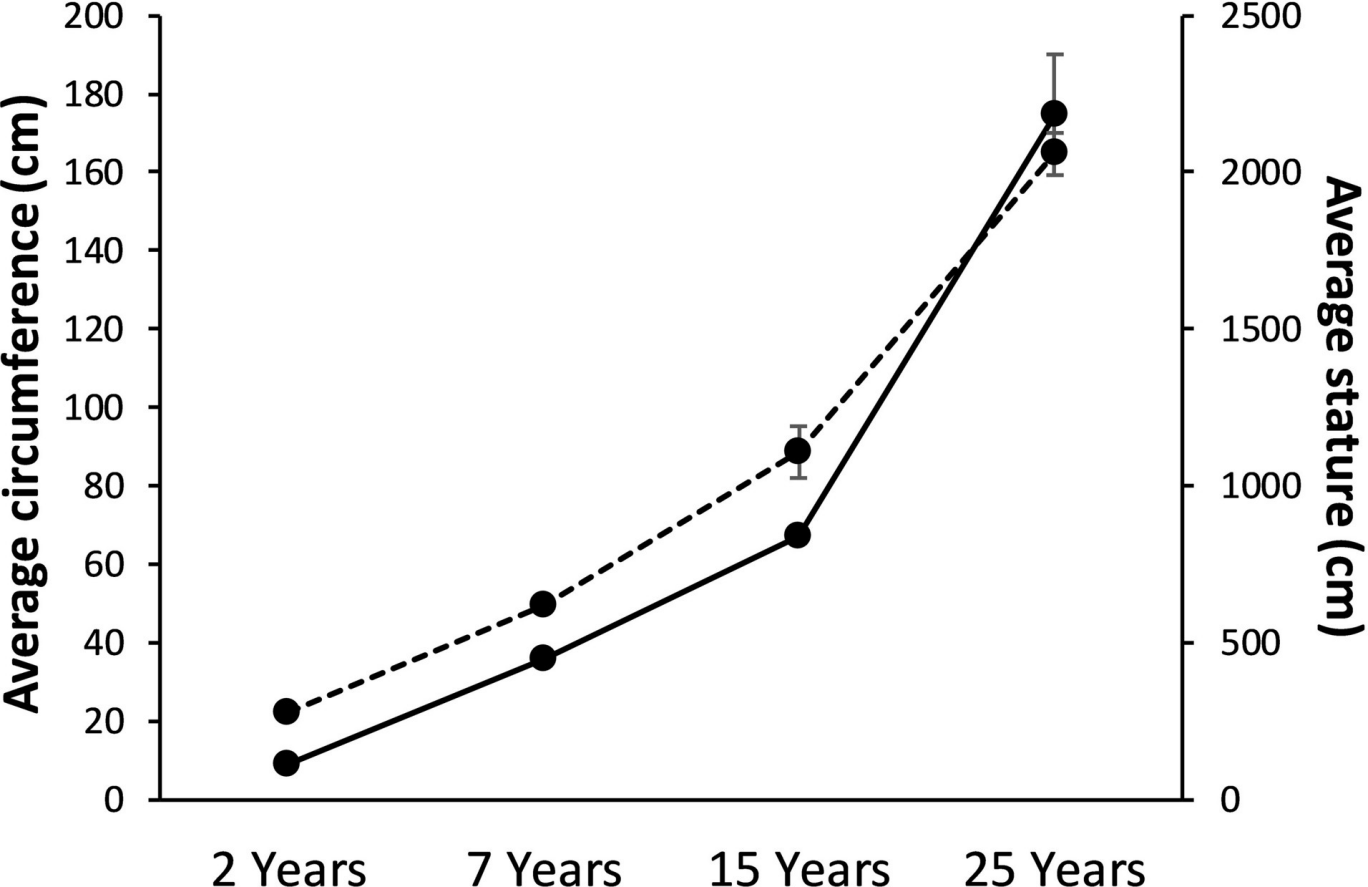
Average circumference (solid line) and stature (dash line) of *Dipterocarpus alatus* at different ages. Data are shown as mean±SD.

Circumference of *Dipterocarpus alatus* aged 2 years had the circumference of 8.78±0.89 cm with the stature of 274±6.54 cm (Fig 1). The circumference and stature had gradually increased and at 25 years of age, their average circumference reached 174.60±15.47 cm with the stature of 2,060±66.93 cm (Fig 1).

### Age-dependent metabolic alterations in leaf and bark

**Fig 2:**
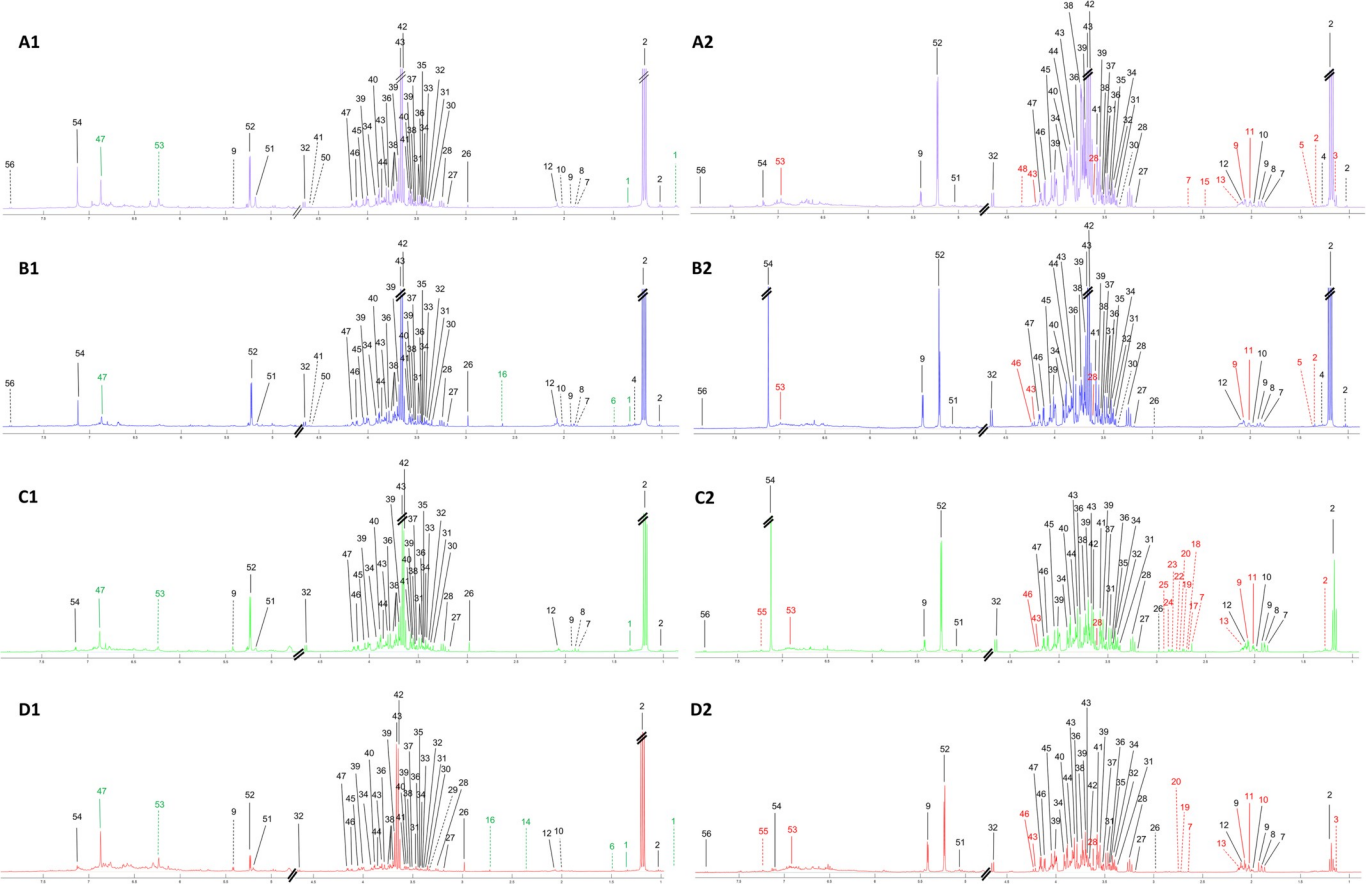
Median standard one-dimensional ^1^H NMR spectra of *Dipterocarpus alatus* at 2 (A), 7 (B), 15 (C) and 25 (D) years of leaf (1) and bark (2). Solid line indicates metabolites found in both leaf and bark at all ages, whereas dashed line indicates metabolites found in either leaf or bark. Green line indicates metabolites found in leaf only, whereas red line indicates metabolites found in bark only. Metabolite list is present in S1 Table in S1 File.

To investigate age-dependent alteration of metabolites in *Dipterocarpus alatus*, ^1^H NMR-based metabolic profiling was performed on both leaf and bark extracts obtained from trees with different ages. A total of 56 metabolites were detected and identified in leaf (Fig 2A) and bark (Fig 2B). Several metabolites including eburnamonine, trans-nerolidol, α-tocopherol, *p*-hydroxyacetophenone, 1-methylnaphthalene, lignin compound 280, lignin compound 3015, lignin compound 292, lignin compound 284 and 2-thiophenecarboxylic acid found in bark, were clearly not seen in leaf (Fig 2).

**Fig 3:**
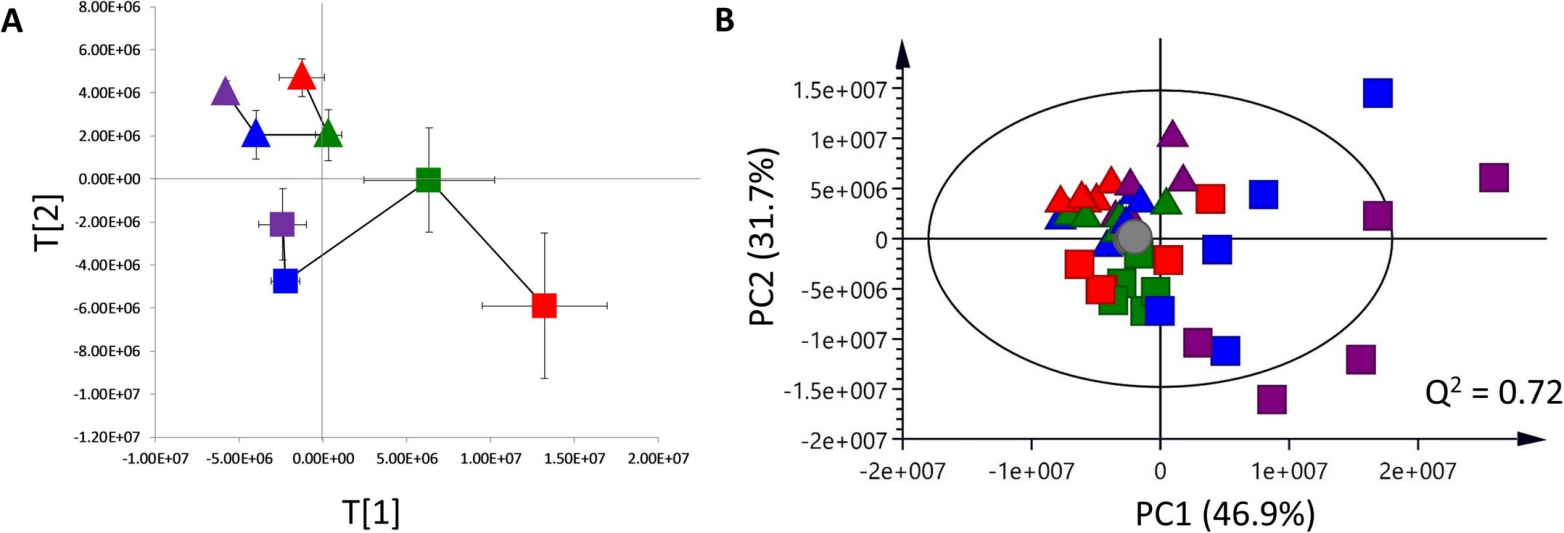
Principal Component Analysis (PCA) of ^1^H NMR spectral data obtained from leaf (triangle) and bark (square) of *Dipterocarpus alatus* at 2 (purple), 7 (blue), 15 (green), 25 (red) years and Quality Control (QC) samples (grey). A: time-trajectory PCA plot. Data are shown as average principal component score±SD. B: PCA score plot of all groups.

Principal component analysis was conducted to visualize the metabolic similarities and differences of leaf and bark metabolome datasets. Leaf at all ages tightly clustered and showed a slight time-related shift in global leaf metabolic profiles over 25 years on PCA trajectory plot (Fig 3A). Different from leaf PCA trajectory plot, bark demonstrated a large degree of variation at different ages ranging from 2 years to 25 years (Fig 3A). A clear separation of trees with different ages was observed along the first principal component (PC1) and the separation between leaf and bark along the second principal component (PC2) (Fig 3B). It is noticeable that 2-year bark group exhibited the strongest variation, followed by 7-year bark group among all samples (Fig 3B).

**Fig 4:**
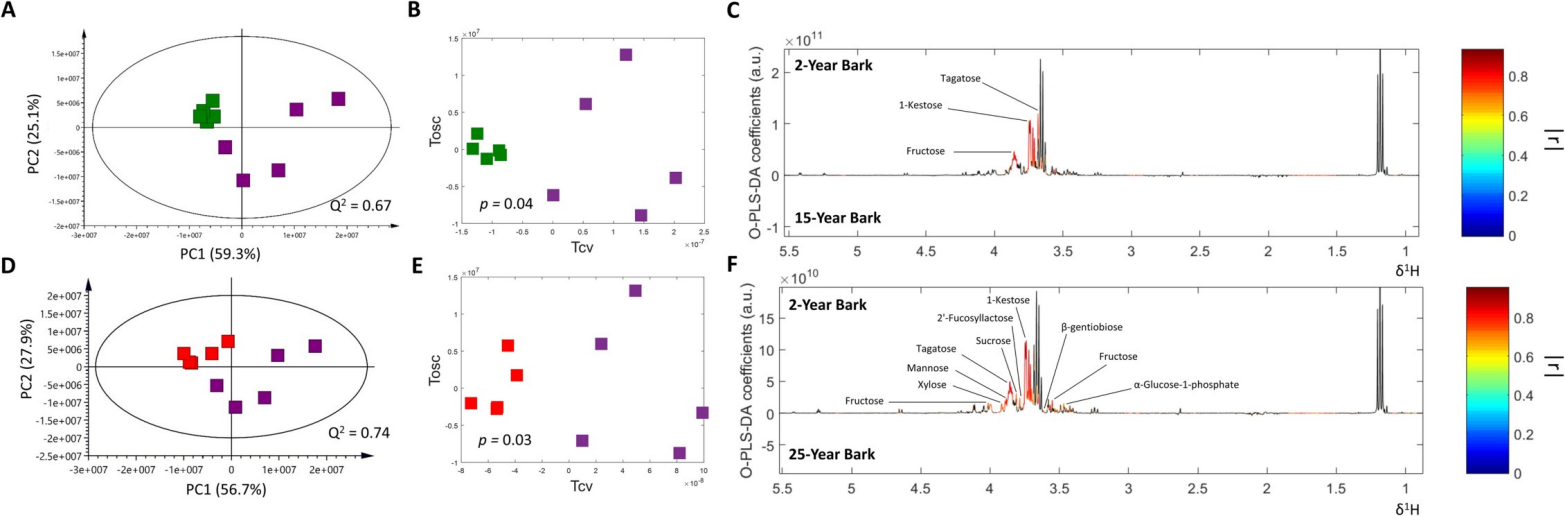
Pairwise PCA and O-PLS-DA comparison models of 2-year bark (purple), 15-year bark (green) and 25-year bark (red). A and D: PCA score plots. B and E: Cross-validated O-PLS-DA score plots. C and F: O-PLS-DA corresponding color-coded correlation coefficient loading plots.

To further extract age-dependent information from leaf and bark metabolome datasets, 16 PCA and O-PLS-DA pairwise comparison models were constructed. PCA and O-PLS-DA pairwise analyses of leaf metabolome did not show significant class discrimination (CV-ANOVA *p* > 0.05) (S1 Table in S2 File). On the contrary, PCA pairwise analysis between 2-year bark and 15-year bark groups clearly showed that they were well separated along PC1 on PCA score plot (Fig 4A). Similarly, a clear class discrimination between 2-year bark and 15-year bark groups was also observed on O-PLS-DA score plot (R^2^X = 90.10%, Q^2^Y = 0.82, CV-ANOVA *p* < 0.05) (Fig 4B). O-PLS-DA corresponding loading plot demonstrated that relative concentrations of fructose, 1-kestose and tagatose were significantly lower in 15-year bark group compared with 2-year bark group (Fig 4C). PCA and O-PLS-DA (R^2^X = 90.90%, Q^2^Y = 0.85, CV-ANOVA *p* < 0.05) score plots of pairwise comparison model between 2-year bark and 25-year bark groups demonstrated similar class separation (Fig 4D and 4E). Moreover, O-PLS-DA corresponding loading plot showed even higher number of metabolites that their relative concentrations significantly decreased in 25-year bark group including fructose, 1-kestose, tagatose, xylose, mannose, sucrose, 2’-fucosyllactose, β-gentiobiose and α-glucose-1-phosphate (Fig 4F).

### Identification of circumference- and stature-associated metabolite fingerprints

**Fig 5:**
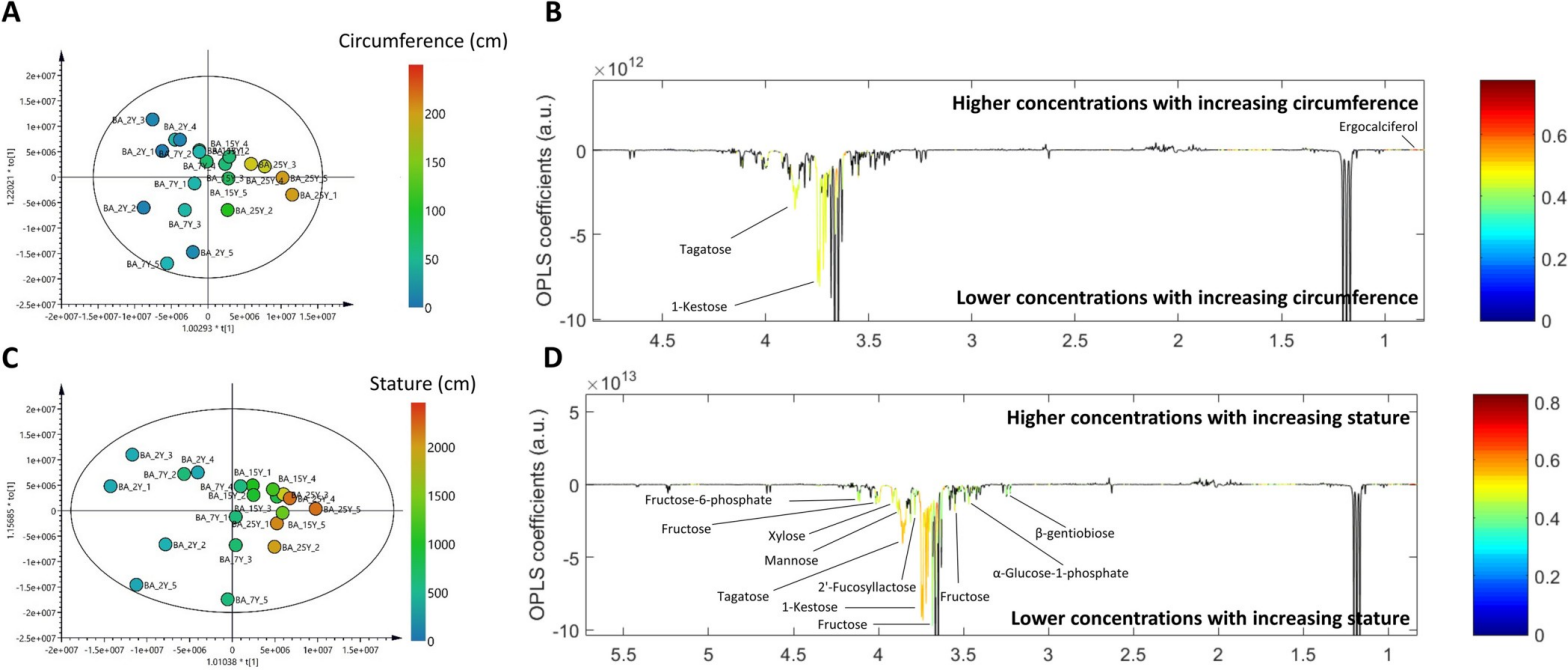
O-PLS regression analyses of bark metabolome data with circumference (A and B) and stature (C and D). A and C: O-PLS score plots. B and D: O-PLS color-coded correlation coefficient loading plots.

^1^H NMR leaf and bark metabolomes were statistically correlated with circumference and stature independently, using *Dipterocarpus alatus* with different ages for O-PLS regression analysis. Interestingly, only bark metabolome demonstrated the promising and valid correlations with circumference and stature (S1 Table in S1 File). Score plot of O-PLS regression model between bark metabolome and circumference (R^2^X = 86.60%, Q^2^Y = 0.57, CV-ANOVA *p* < 0.05) showed prominent class clustering (Fig 5A). O-PLS loading plot exhibited that relative concentrations of tagatose and 1-kestose in bark of *Dipterocarpus alatus* significantly decrease with increasing circumference (Fig 5B). In addition to reduced concentrations of 1-kestose and tagatose, O-PLS regression model between bark metabolome and stature demonstrated a greater metabolic change with increasing stature (Fig 5C and 5D). Relative concentrations of fructose, fructose-6-phosphate, xylose, mannose, 2’-fucosyllactose, α-glucose-1-phosphate, β-gentiobiose, 1-kestose and tagatose were shown to be significantly lower with increasing stature (Fig 5D) that demonstrates the similar metabolic fingerprints to the pairwise comparison model between 2-year bark and 25-year bark groups. However, it is noteworthy that only levels of tagatose and 1-kestose were significantly reduced when compared with increasing circumference.

**Fig 6:**
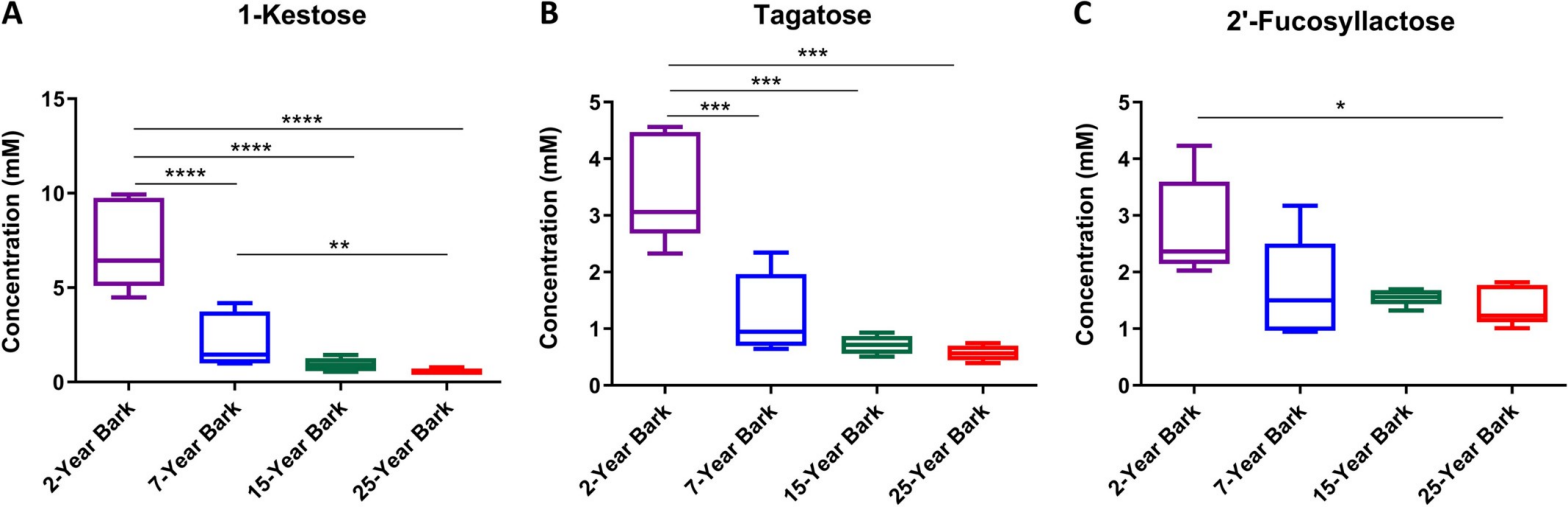
Multiple comparisons of absolute concentrations of selected O-PLS significant metabolites in bark using Tukey’s multiple comparison test. *, **, *** and **** indicate significant levels of metabolite differences at adjusted *p* < 0.05, *p* < 0.01, *p* < 0.001 and *p* < 0.0001, respectively.

Maximum intensity of all significant metabolites was used for quantification of absolute concentrations in regards with TSP as the internal standard with known concentration. Of nine bark metabolites, our findings showed that 1-kestose, and tagatose significantly decreased on age-dependent manner (Fig 6A and 6B). Two-year bark group contained average 1-kestose concentration of 7.22±1.97 mM and its concentration decreased to 2.18±1.31, 0.92±0.30 and 0.55±0.15 mM in 7-, 15- and 25-year bark groups, respectively (Fig 6A). Average concentration of tagatose contained in 2-year bark was 3.47±0.78 mM before decreasing to 1.25±0.64, 0.71±0.14 and 0.57±0.12 mM in bark of 7, 15 and 25 years of age, respectively (Fig 6B). Although 2’-fucosyllactose did not exhibit such the similar changes, its concentration was found to be remarkably decreased when comparing 2-year bark (2.77±0.73 mM) with 25-year bark (1.45±0.34 mM) groups (Fig 6C).

## Discussion

*Dipterocarpus alatus* is very well known for its use of oleoresin; however, its global chemical baseline information at different ages is still inadequate. In addition, age determination using the traditional dendrochronology which is difficult and inaccurate as *Dipterocarpus alatus* has thick wood and enriched oleoresin that may cause the interference to the visualization of annual growth rings. In the current study, we have, for the first time, reported the chemical baseline characteristics at different ages including global metabolic profiles of bark and leaf, together with their age-, circumference- and stature-associated metabolite fingerprints of *Dipterocarpus alatus*. We have globally profiled the metabolites obtained from bark and leaf of *Dipterocarpus alatus* using ^1^H NMR spectroscopy-based metabolomics approach. We demonstrated that there is a total of 56 metabolites shared between bark and leaf with different concentrations. Our findings also indicate the remarkable metabolic shift in bark of *Dipterocarpus alatus* over time. From our finding, several metabolites including eburnamonine, trans-nerolidol, α-tocopherol, *p*-hydroxyacetophenone, 1-methylnaphthalene, lignin compound 280, lignin compound 3015, lignin compound 292, lignin compound 284 and 2-thiophenecarboxylic acid found in bark, were clearly not seen in leaf. Lignin is an important component of wood, particularly perennials. It helps strengthen the plants and enable their waterproof and disease-resistant properties [20]. Moreover, lignin is remarkably involved in cell wall lignification which is a complex process occurring exclusively in higher plants; its main function is to strengthen the plant vascular body [21].

Principal component analysis was conducted to visualize the metabolic similarities and differences of leaf and bark metabolome datasets. Our findings demonstrated that leaf at all ages tightly clustered and a slight time-related shift in global leaf metabolic profiles was evident, whereas bark demonstrated a larger degree of variation at different at different ages ranging from 2 years to 25 years. The minute shift of leaf metabolome at different ages may result from the fact that the main function of the leaf is photosynthesis which is a continual life-time process to convert light energy into chemical energy in the form of nutrients [22, 23]. The major products obtained from such autotrophic process are sugars that can be further converted in other essential compounds, for example, starch accumulated in plant tissue [24]. This suggests that biosynthesis, rather than biodegradation, is mainly involved in the functions of leaf enabling more stable metabolic compositions compared to other parts of plants that is consistent with the results of time-trajectory principal component analysis of leaf datasets shown similarities in global leaf metabolic profiles. In contrast, the phloem which occurs in bark plays an essential role in transporting photosynthetic products from the leaf to other parts of the plant and involved in the defense of tree [25] that corresponds to the large degree of variation in metabolic profiles obtained from bark at different ages.

In regards with PCA and O-PLS-DA pairwise analyses of leaf metabolome, there was no significant class discrimination while bark metabolic profiles at different ages demonstrated that relative concentrations of fructose, 1-kestose and tagatose were significantly lower in 15-year and 25-year bark group when compared with 2-year bark group. 1-kestose and tagatose are metabolites derived from sucrose biosynthesis pathway [26]. Sucrose is a disaccharide, which consists of a single molecule of two types of monosaccharide, fructose and glucose. Sucrose influences plant size by promoting cell division in apical meristem [27, 28] and is an important product found in photosynthesis that is a signal to control growth and differentiation occurring in areas with chlorophyll [27, 29]. Products from photosynthesis will be stored at different parts of the plant and some plants stored in the trunk [29]. For these reasons, the decrease in 1-kestose, tagatose and fructose concentrations in bark of *Dipterocarpus alatus* over time can be caused by the use of these substances in growth and differentiation [30]. We further demonstrated that ^1^H NMR leaf and bark metabolomes were statistically correlated with circumference and stature independently. Interestingly, only bark metabolome demonstrated the promising and valid correlations with circumference and stature. Our findings showed that relative concentrations of tagatose and 1-kestose in bark of *Dipterocarpus alatus* significantly decreased with rising circumference and stature that together support the similar metabolic fingerprints of the pairwise comparison model between 2-year bark and 25-year bark groups as previously aforementioned. The current exploratory study sheds light on the novel method for determining age and growth of *Dipterocarpus alatus* that provides more precise and less cost-effective analytical platform. Though the present protocol relies on the high-throughput instrument, the results of changing metabolites used as biological indicators can be further adopted in developing more portable and more convenient on-site biosensor.

## Conclusion

Knowing the precise age of *Dipterocarpus alatus* is very important as it helps convey the appropriate use and reduce the chance of too early falsified harvest which may lead to the destruction and waste of trees. Bark metabolome significantly differed from leaf metabolome which showed less degree of metabolic alterations with increasing age. Significantly decreasing levels of certain metabolites including tagatose, 1’kestose and 2’-fucosyllactose exhibited the promising patterns that can be employed as the novel biological indicators for determining age and growth of *Dipterocarpus alatus*.

## Supporting information

S1 File The metabolite assignment from ^1^H NMR analysis. (DOCX)

S2 File Multivariate statistical analysis of bark and leaf metabolome data. (DOCX)

S1 Raw data (RAR)
