# Supplementary material for: 1H NMR metabolic phenotyping of Dipterocarpus alatus as a novel tool for age and growth determination
Source: PLoS One. 2020 Dec 15;15(12):e0243432. doi: 10.1371/journal.pone.0243432 (PMC7737897; doi:10.1371/journal.pone.0243432)
Supplement: S1 File — (DOCX) [file pone.0243432.s001.docx]

**S1 File. The metabolite assignment from ^1^H NMR analysis**

**S1 Table** The identification of metabolites in the studied plant extracts. ^1^H NMR data are measured in ppm.

| **No** | **Metabolites** | **Chemical shift** | **BMRB ID** |
| --- | --- | --- | --- |
| 1 | Ergocalciferol | 0.8541(d), 6.232(m), 6.871(s) | bmse000510 |
| 2 | 17-Acetyl-3,17-dihydroxy-10,13-dimethyl-2,3,4,5,6,7,8,9,12,14,15,16-dodecahydro-1H-cyclopenta[a]phenanthren-11-one | 1.184(t), 1.340(t), 3.647(q) | bmse001170 |
| 3 | Stigmasterol | 1.136(s) | bmse000528 |
| 4 | Diosgenin | 1.278(t) | bmse001247 |
| 5 | Eburnamonine | 1.383(d), 2.223(s), 7.417(bs) | bmse001153 |
| 6 | Pomiferin | 1.476(d) | bmse001284 |
| 7 | Epsilon-caprolactone | 1.867(t), 2.696(s) | bmse000493 |
| 8 | *N*-acetyl-D-Phenyl alanine | 1.901(t), 2.696(s), 4.152(q) | bmse000479 |
| 9 | (E)-3,7,11,15-tetramethylhexadec-2-en-1-ol | 1.928(t),2.062(m), 2.696(s), 4.152(q), 4.445(s) | bmse001123 |
| 10 | Dihydroisorescinnamine | 2.014(m) | bmse001300 |
| 11 | trans-Nerolidol | 2.028(m) | bmse001254 |
| 12 | Pyrethrosin | 2.094(m) | bmse001219 |
| 13 | (+)Alpha-tocopherol | 2.109(m) | bmse000600 |
| 14 | Hordenine | 2.353(s) | bmse001239 |
| 15 | p-Hydroxyacetophenone | 2.474(s), 4.445(m) | bmse000670 |
| 16 | 3-Acetylphenylboronic acid | 2.627(s), 7.485(d) | bmse000960 |
| 17 | Lignin_cw_compound_280 | 2.677(s), 2.832(d) | bmse010187 |
| 18 | 1-Methylnaphthalene | 2.696(s), 2.724(d), 2.840(t) | bmse000531 |
| 19 | Pubescine | 2.724(d), 2.763(s) | bmse001220 |
| 20 | 4-Hydroxy-2,5-dimethylfuran-3-one | 2.751(s), 2.715(s), 4.472(s) | bmse001203 |
| 21 | L-Asparagine | 2.763(s), 2.893(bs) | bmse000030 |
| 22 | Lignin_cw_compound_3015 | 2.80(s) | bmse010340 |
| 23 | Seco-isolariciresinol | 2.832(d) | bmse010322 |
| 24 | Quinidine | 2.872(s) | bmse000511 |
| 25 | Lignin_cw_compound_3004 | 2.932(s) | bmse010333 |
| 26 | 17-Alpha-hydroxyprogesterone | 2.981(s) | bmse000472 |
| 27 | Acetylcarnitine | 3.185(s) | bmse000142 |
| 28 | Beta-gentiobiose | 3.246(t), 3.377(m), 3.468(d), 3.514(m), 3.615(s), 3.70(m), 3.785(s), 3.811(m), 3.916(s), 3.993(d), 4.045(s), 4.111(d), 4.637(d), 5.229(d) | bmse000313 |
| 29 | Scyllo-Inositol | 3.331(s) | bmse000113 |
| 30 | Formaldehyde | 3.349(s) | bmse000256 |
| 31 | Alpha-D-glucose-1-phosphate | 3.377(m), 3.402(m), 3.468(d), 3.70(m), 3.785(s), 3.811(m), 3.917(s), 3.993(d), 4.045(s), 3.4111(d), 4.637(d), 5.229(d) | bmse000086 |
| 32 | D-Glucose | 3.402(m), 3.468(d), 3.514(m), 3.579(m), 3.615(s), 3.785(s), 3.811(m), 3.917(s), 3.997(d), 4.044(s), 4.111(d), 4.637(d), 5.229(d) | bmse000855 |
| 33 | D-Cellobiose | 3.411(m), 3.468(d), 3.514(m), 3.615(s), 3.70(m), 3.786(s), 3.811(m), 3.916(s), 3.997(d), 4.044(s), 4.111(d), 4.637(d), 5.229(d) | bmse000138 |
| 34 | D-Xylose | 3.424(m), 3.468(d), 3.514(m), 3.615(s), 3.70(m), 3.786(s), 3.811(m), 3.916(s), 3.997(d), 4.044(s), 4.111(d), 4.637(d), 5.229(d) | bmse000026 |
| 35 | D-Psicose | 3.435(m), 3.468(d), 3.514(m), 3.615(s), 3.70(m), 3.786(s), 3.811(m), 3.916(s), 3.997(d), 4.044(s), 4.111(d), 4.637(d), 5.229(d) | bmse000965 |
| 36 | 2'-Fucosyllactose | 3.448(m), 3.468(d), 3.514(m), 3.615(s), 3.70(m), 3.786(s), 3.811(m), 3.916(s), 3.997(d), 4.637(d) | bmse001326 |
| 37 | D-Glucose 6-phosphate | 3.514(m), 3.615(s), 3.70(m), 3.786(s), 3.811(m), 3.916(s), 3.997(d), 4.044(s), 4.111(d), 4.637(d), 5.229(d) | bmse000086 |
| 38 | 1-Kestose | 3.527(m), 3.615(s), 3.70(m), 3.786(s), 3.811(m), 3.916(s), 3.997(d), 4.044(s), 4.111(d), 4.637(d), 5.229(d) | bmse001112 |
| 39 | D-Fructose | 3.55(m), 3.615(s), 3.70(m), 3.786(s), 3.811(m), 3.916(s), 3.997(d), 4.044(s), 4.111(d), 4.152(q), 4.637(d), 5.229(d) | bmse000010 |
| 40 | D-Mannose | 3.566(m), 3.615(s), 3.70(m), 3.786(s), 3.811(m), 3.916(s), 3.997(d), 4.044(s), 4.111(d), 4.152(q), 4.637(d) | bmse000018 |
| 41 | Lacto-*N*-fucopentaitoll | 3.579(m), 3.615(s), 3.70(m), 3.786(s), 3.811(m), 3.916(s), 3.997(d), 4.044(s), 4.111(d), 4.152(q), 4.637(d), 5.229(d) | bmse001328 |
| 42 | 3-Fucosyllactose | 3.647(q) | bmse001330 |
| 43 | Sucrose | 3.665(q) | bmse000119 |
| 44 | D-Tagatose | 3.858(m), 3.915(s), 3.997(d), 4.044(s), 4.111(s) | bmse000023 |
| 45 | D-Ribulose 5-phosphate | 4.045(s), 4.111(d), 4.152(q), 4.637(d), 5.229(d) | bmse000278 |
| 46 | D-Fructose-6-phosphate | 4.111(d), 4.637(d) | bmse000012 |
| 47 | N-Acetyl-L-Glutamine | 4.152(q) | bmse000145 |
| 48 | Lignin_cw_compound_292 | 4.349(q) | bmse010199 |
| 49 | Lignin_cw_compound_284 | 4.445(m) | bmse010191 |
| 50 | D-Galactose | 4.637(d), 5.229(d) | bmse000013 |
| 51 | Phosphoenolpyruvic acid | 5.173(s) | bmse000107 |
| 52 | L-Arabinose | 5.229(d) | bmse000213 |
| 53 | Lignin_cw_compound_2004 | 6.232(s), 6.87(s) | bmse010458 |
| 54 | Medicarpin | 7.128(s) | bmse001290 |
| 55 | 2-Thiophenecarboxylic acid | 7.151(s) | bmse000848 |
| 56 | 4-Methoxy benzaldehyde | 7.852(d) | bmse010130 |

Abbreviations: s: singlet; bs: broad singlet; d: doublet; t: triplet; q: quartet; m: multiplet
