# Supplementary material for: 1H NMR metabolic phenotyping of Dipterocarpus alatus as a novel tool for age and growth determination
Source: PLoS One. 2020 Dec 15;15(12):e0243432. doi: 10.1371/journal.pone.0243432 (PMC7737897; doi:10.1371/journal.pone.0243432)
Supplement: S2 File — (DOCX) [file pone.0243432.s002.docx]

**S2 File. Multivariate statistical analysis of bark and leaf metabolome data**

**S1 Table** The multivariate statistical analysis models

| **Model no.** | **Comparison** | **PCA** | | | **O-PLS-DA** | | | | **O-PLS** | | | |
| --- | --- | --- | --- | --- | --- | --- | --- | --- | --- | --- | --- | --- |
|  |  | *R^2^* | | *Q^2^* | *R^2^X* | | *Q^2^Y* | CV-ANOVA  p-value | *R^2^X* | | *Q^2^Y* | CV-ANOVA  p-value |
|  |  | PC1 | PC2 |  | t[1] | to[1] |  |  | t[1] | to[1] |  |  |
| 1 | All data groups | 46.90% | 31.70% | 0.72 |  |  |  |  |  |  |  |  |
| 2 | Leaf all ages | 44.90% | 26.00% | 0.64 |  |  |  |  |  |  |  |  |
| 3 | Bark all ages | 52.00% | 28.50% | 0.74 |  |  |  |  |  |  |  |  |
| 4 | 2-Year Leaf vs. 7-Year Leaf | 52.80% | 24.40% | 0.44 | 61.00% | 90.30% | 0.37 | 0.61 |  |  |  |  |
| 5 | 2-Year Leaf vs. 15-Year Leaf | 46.50% | 27.80% | 0.54 | 78.00% | 97.30% | 0.2 | 0.94 |  |  |  |  |
| 6 | 2-Year Leaf vs. 25-Year Leaf | 53.20% | 26.30% | 0.62 | 76.70% | 84.40% | 0.54 | 0.34 |  |  |  |  |
| 7 | 7-Year Leaf vs. 15-Year Leaf | 55.40% | 16.70% | 0.54 | 76.30% | 86.00% | -1.05 | 1 |  |  |  |  |
| 8 | 7-Year Leaf vs. 25-Year Leaf | 50.20% | 28.10% | 0.58 | 81.30% | 94.60% | 0.19 | 0.95 |  |  |  |  |
| 9 | 15-Year Leaf vs. 25-Year Leaf | 52.20% | 19.20% | 0.58 | 71.00% | 87.60% | 0.62 | 0.22 |  |  |  |  |
| 10 | 2-Year Bark vs. 7-Year Bark | 57.20% | 24.80% | 0.7 | 86.00% | 93.80% | 0.58 | 0.47 |  |  |  |  |
| 11 | 2-Year Bark vs. 15-Year Bark | 59.30% | 25.10% | 0.67 | 83.90% | 90.10% | 0.82 | **0.04** |  |  |  |  |
| 12 | 2-Year Bark vs. 25-Year Bark | 56.70% | 27.90% | 0.74 | 84.30% | 90.90% | 0.85 | **0.03** |  |  |  |  |
| 13 | 7-Year Bark vs. 15-Year Bark | 65.00% | 17.80% | 0.63 | 82.50% | 32.80% | 0.65 | 0.18 |  |  |  |  |
| 14 | 7-Year Bark vs. 25-Year Bark | 59.60% | 22.50% | 0.65 | 86.80% | 92.20% | 0.26 | 0.91 |  |  |  |  |
| 15 | 15-Year Bark vs. 25-Year Bark | 47.80% | 23.00% | 0.43 | 77.80% | 88.30% | 0.18 | 0.91 |  |  |  |  |
| 16 | O-PLS Bark all ages vs. circumference |  |  |  |  |  |  |  | 83.00% | 86.60% | 0.57 | **0.05** |
| 17 | O-PLS Leaf all ages vs. circumference |  |  |  |  |  |  |  | 76.30% | 69.60% | 0.33 | 0.43 |
| 18 | O-PLS Leaf all ages vs. stature |  |  |  |  |  |  |  | 75.90% | 74.40% | 0.32 | 0.45 |
| 19 | O-PLS Bark all ages vs. stature |  |  |  |  |  |  |  | 80.00% | 67.70% | 0.59 | **0.005** |
